# Empowerment of Primary Healthcare Providers on the Prevention and Management of Dental or Oral Health Issues among Postchemotherapy Patients in Pandemic

**DOI:** 10.1155/2022/9087776

**Published:** 2022-03-10

**Authors:** Premalatha Paulsamym, Krishnaraju Venkatesan, Jamal Moideen Muthu Mohamed, ShadiaHamoud Alshahrani, Ramasubbamma Ramaiah, Vigneshwaran Easwaran, Mohamed El-Sherbiny, Mohamed Dosoky, Noohu Abdulla Khan, Kousalya Prabahar, Geetha Kandasamy, Kibebe Sahile

**Affiliations:** ^1^College of Nursing, Mahalah Branch for Girls King Khalid University, Abha, Asir, Saudi Arabia; ^2^Department of Pharmacology, College of Pharmacy, King Khalid University, Abha, Asir, Saudi Arabia; ^3^College of Pharmacy, Shri Indra Ganesan Institute of Medical Science, Tiruchirappalli 620012, Tamil Nadu, India; ^4^Department of Clinical Pharmacy, College of Pharmacy, King Khalid University, Abha, Saudi Arabia; ^5^Department of Basic Medical Sciences, College of Medicine, AlMaarefa University, 71666 Riyadh, Saudi Arabia; ^6^Department of Neuroscience Technology-College of Applied Sciences, Jubail Imam Abdulraman Bin Faisal University, Al Jubail, Saudi Arabia; ^7^Department of Pharmacy Practice, Faculty of Pharmacy, University of Tabuk, Tabuk 71491, Saudi Arabia; ^8^Department of Chemical Engineering, College of Biological and Chemical Engineering, Addis Ababa Science and Technology University, Addis Ababa, Ethiopia

## Abstract

**Objectives:**

The study aim was to evaluate the empowerment of primary healthcare providers on the prevention and management of dental or oral health issues among postchemotherapy (PC) patients, in selected rural regions, India, during a pandemic.

**Methods:**

Initially, 240 PHPs were recruited by convenient and snow ball sampling with 90.3% response rate. A descriptive, cross-sectional study was adopted using a self-administered questionnaire with 5 sections: demographics, identification of dental/oral health issues, knowledge, attitude, and practice on prevention and management of dental/oral health problems in PC patients. Statistical Packages for Social Sciences (SPSS) version 23.0 was used for statistical analysis.

**Results:**

The overall knowledge was better among nurses (64.56%), followed by pharmacists (54.5%). 81.65% of PHPs were willing to learn more and expressed the need for collaboration with dentists. In the past 3 months, 18.81% of them had PC patients with dental/oral health issues, but only 3.5% of nurses and 0.8% of pharmacists treated them. The logistic regression model revealed higher scores in mucositis/mucosal pain (OR = 1.41), altered taste sensation (OR = 1.34), sensitive gums (OR = 1.71), and dental caries (OR = 1.32) domains (*p* < 0.05). Those who had readiness to learn (OR = 5.37), nurses and pharmacists, and having less years of experience (OR = 1.31) and higher degree (OR = 1.4) had a positive attitude (*p* < 0.05).

**Conclusion:**

PHPs had limited empowerment in terms of knowledge and practice but showed a positive attitude toward the prevention and management of dental/oral health issues of PC patients. For better practice, continuing education and collaboration with dental professionals is essential.

## 1. Introduction

Cancer is the second leading cause of mortality in developed countries, trailing only cardiovascular diseases. It is still a major public health concern. Oncology management encompasses a wide range of treatments addressing the preventive, curative, and rehabilitative aspects of cancer. Chemotherapy is one of the most effective treatment options [1]. Advances in cancer treatment have resulted in the longer survival of these patients. Nonetheless, the side effects of cancer therapies continue to be a major concern, as they may limit the effectiveness of the treatment and have a negative impact on the quality of life both during and after therapy.

The extent of chemotherapeutic agents' oral toxicity appears to be related to the dose and frequency of administration. They can cause direct damage to the soft and hard tissues of the oral structures, and their systemic toxicity can cause indirect damage. These oral complications, whether acute or chronic, can occur during or after cancer treatment. Many drugs target rapidly proliferating cells; however, they also have the same effect on rapidly proliferating normal tissues, such as the oral mucosa, and destroy the mucosal layer's basal cells. Their replacement or turnover is hampered, resulting in mucosal ulceration and decreased salivary function [2–4]. There is an indirect toxic effect that is caused outside the oral cavity such as myelosuppression or the destruction of immune cells. In general, more than 30–35% of cancer patients will experience mucositis, altered taste, xerostomia, sensitive gums, dental caries, speaking difficulties, and jaw pain [5, 6]. These side effects can have an implication on the patients' quality of life and as a whole clinical outcome.

The Global Burden of Disease Study (2017) reported that oral diseases affect 3.5 billion people worldwide. The prevalence of dental caries in patients who undergo chemotherapy was 37.3%. The oral cavity is extremely vulnerable to the direct and indirect toxic effects of chemotherapy due to the high cellular turnover rate, microflora, and oral tissue trauma associated with daily oral functions [7]. The estimated prevalence of oral complications ranges from 31 to 93% [8].

Effective oral health management prior to, during, and after treatment is therefore critical to improving patient well-being [9]. Each health professional engaged in the cancer care continuum ought to be aware of such specific oral health adverse reactions and be able to give the necessary precautions to avoid them. In 2020, an enigmatic virus known as SARS-CoV-2 confronted the medical community and wreaked havoc all over the world. According to the recently published data, COVID-19 patients have been prioritized over other patients, including cancer patients, due to the unforeseen conditions of the pandemic. The COVID-19 pandemic had a wide-ranging impact on healthcare services, beginning with disrupting regular patient flow to treatment centers, stressing and intimidating healthcare resources, and leading to the deployment of additional protective measures and social distancing with increased use of telehealth and virtual medicine. Access to healthcare and clinical services became difficult for cancer patients as well during the COVID-19 pandemic [10].

Due to the above-mentioned unprecedented circumstances, dental therapies have been halted in a lot of countries due to the role of saliva and aerosols in the spread of COVID-19. This has resulted in a disruption in the allocation of dental care to all patients, including postchemotherapeutic patients, for whom it is absolutely vital [11]. More importantly, postchemotherapeutic patients must have oral re-examinations every month for the first three months, then once in three months during the first year, and then every six months for the next three years. In this situation, the role of primary healthcare centers (PHCs) in providing adequate support for postchemotherapeutic oral ailments is critical.

In fact, in developing countries such as India, PHCs represent the first tier in healthcare system, providing a range of essential outpatient services to people living in the rural, suburban, and hard-to-reach areas. During the COVID-19 pandemic, the preparedness of PHCs in providing safe, patient-centered care and meeting the current health needs of the population is crucial. Hence, determining the empowerment of primary healthcare providers (PHPs) in terms of knowledge, attitude, and practice on the prevention and management of dental or oral health issues among postchemotherapy (PC) patients is the need of the hour.

## 2. Methods

### 2.1. Research Approach and Design

The research approach is quantitative. A descriptive, cross-sectional study was adopted for determining the empowerment of Primary Healthcare Providers (PHPs) in terms of knowledge, attitude, and practice on the prevention and management of dental or oral health issues among postchemotherapy (PC) patients.

### 2.2. Population and Setting

PHPs working in the rural regions of Tamil Nadu, India, were the population of the study. Three rural districts were selected and PHPs working in PHCs and subcenters were selected as samples for the study.

### 2.3. Sample Size and Sampling Process

The nonprobability convenient sampling technique was adopted to obtain samples from the three rural districts of Tamil Nadu, India.

The minimum suggested sample size was computed by Raosoft, an online sample size calculator having the overall population (approx. 681 PHPs) of three districts, a 95% confidence with margin of error as 5%, and the expected response rate of 60%. The calculated sample size was 240. Initially, 3 PHCs of each district were visited by one of the investigators personally to select the samples and the snow ball sampling technique was used to contact the rest of the participants. Registered nurses, pharmacists, and laboratory technicians who were willing to participate in the study were included in the study. PHPs who had basic professional training of less than 2 years were excluded from the study.

### 2.4. Data Collection Tools/Instruments

The self-administered tool consisted of 4 sections.

#### 2.4.1. Section 1

Section 1 describes the demographic data of the participants.

#### 2.4.2. Section 2

Section 2 describes a checklist for the identification of dental/oral health issues of postchemotherapeutic patients.

#### 2.4.3. Section 3

Section 3 describes knowledge on the prevention and management of dental/oral health problems of postchemotherapeutic patients. Among the dental and oral health problems, the most common and vital issues, such as mucositis/mucosal pain, dysgeusia/hypogeusia/altered taste sensation, xerostomia/dry mouth/hyposalivation, sensitive gums, and dental caries, were included in the knowledge questionnaire. It included 15 multiple-choice questions and each question had the score of “1” for the correct answer and “0” for the wrong answer.

#### 2.4.4. Section 4

The attitude of the PHC providers toward the prevention and management of dental/oral health problems was assessed in this section. Participants assessed their attitude by themselves on a 5-point scale, with strongly agree − 5 to strongly disagree − 1. The participants' attitude was assessed by the mean scores of the 5 statements.

#### 2.4.5. Section 5

The practice of PHC providers toward the prevention and management of dental/oral health problems of PC patients was evaluated with seven “yes” or “no” questions.

### 2.5. Reliability and Validity

The content validity of the tool was obtained from five experts in Nursing and one general physician and a Dental Specialist. The reliability of the tool was assessed by a pilot study by Cronbach's Alpha test (internal consistency) and the *r* value is 0.813, which was found to be highly reliable. The data were collected from the participants by electronic media such as WhatsApp, e-mail, and Instagram from February to April 2021. The PHPs were circulated with a Google Docs questionnaire and reminders were given thrice to complete and return the docs. Out of the 240 PHPs, 218 (90.8%) participants returned the completed tool.

### 2.6. Ethical Consideration

Official permission from the Medical Directors of the PHCs as well as ethical permission from the Institutional Ethical Committee with IEC/LCN/2021 was obtained. Consent from the participants was collected before starting the study by explaining the aim of the study, their role, confidentiality of the information, and their right to depart from the study at any point of data collection. The participants were given a soft copy of the study material after the data collection period in the form of a power point presentation on the prevention and management of dental/oral health among postchemotherapy patients for enhancing their knowledge on the same.

### 2.7. Statistical Analysis

The data were processed and analyzed by the SPSS software using descriptive and inferential statistics. Descriptive statistics, *χ*^2^ tests (for categorical variables), and regression analysis were used to compare knowledge, attitude, and practice with demographics by SPSS 23.0 for Mac. *p* < 0.05 was considered to be significant in this study.

## 3. Results

### 3.1. Demographic Data of the Study Participants

The collected data from 218 PHPs were tabulated and the sociodemographic features of the study participants were tabulated in Table 1. Out of 240 PHPs, 218 responses were received with a response rate of 90.83%. Most of the participants (63.3%) were females and 23.4% of the participants were aged between 20 and 25 years. About 51.38% were nurses, 32.57% were pharmacists, and 16.05% were lab technicians. Among the PHPs, almost half (48.17%) of them had a diploma degree, and a small minority (6.42%) held postgraduate qualifications. Their work experience was variable, ranging from less than one year to greater than 10 years. More than half (53.67%) of them were getting 50 to 100 patients per day and more than two-thirds (77.99%) do not get any patients with postchemotherapy treatment. Among the PHPs, 18.81% of them had treated PC patients with dental or oral health issues.

### 3.2. PHPs' Knowledge on Dental- and Oral-Related Health Issues among PC Patients

Among the PHPs, most of them (88.53%) identified cachexia as one the major health issues among postchemotherapy patients followed by dental caries (85.78%). The least identified problem was mastication/jaw pain(26.15%) (Table 2).

The overall level of knowledge on dental or oral health was better among nurses (64.56%), followed by pharmacists (54.5%); lab technicians scored less (30.03%) comparatively. However, pharmacists had better knowledge (65.2% vs 63.0% and 27.7%) on mucositis/mucosal pain among the PHPs (Table 3).

### 3.3. PHPs' Attitude on Dental- and Oral-Related Health Issues among PC Patients

**Figure 1:**
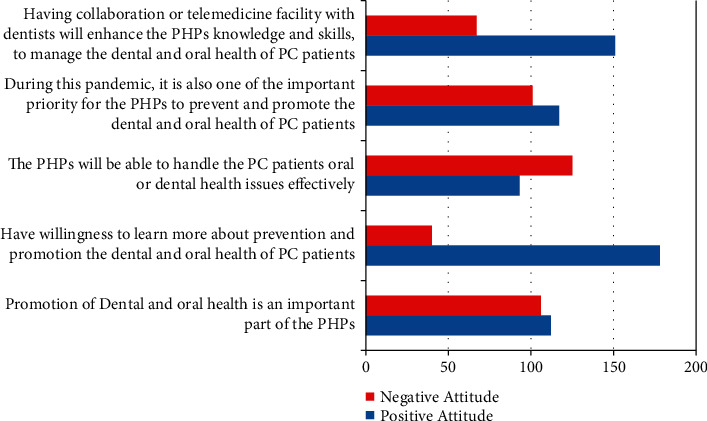
PHPs' attitude toward the dental/oral healthcare among PC patients.

Figure 1 shows the PHPs' attitude toward the dental/oral healthcare among PC patients. Among these, 178 PHPs expressed positively of their willingness to learn more and also opined that having collaboration or telemedicine facility with dentists will enhance the PHPs' knowledge and skills to manage the dental/oral health of PC patients (151 PHPs). About the negative attitude, 125 PHPs had a negative opinion to handling the PC patients' dental/oral health issues effectively. This may occur due to inadequate knowledge and practice experience while handling PC patients with oral health problems.

### 3.4. PHPs' Practice on Prevention and Management of Dental and Oral Health Problems among PC Patients

Determining the practice of PHPs toward the prevention and management of dental/oral health problems of PC patients reveals that though 18.81% of them had PC patients with dental/oral health issues in the past 3 months, 65.1% of the nurses, 71.4% of the pharmacists, and 89.3% of the lab technicians never treated or counselled the PC patients (Table 4). Only 3.5% of the nurses and 0.8% of the pharmacists treated PC patients dental/oral issues often.

### 3.5. Multivariate Analysis

All variables were used in their continuous form except for profession, readiness to learn, and qualification. Goodness of fit with the Hosmer and Lemeshow test, (HL test) *p*- value = 0.13.

After controlling the demographic data of the PHPs, the logistic regression model revealed higher scores in the mucositis/mucosal pain (OR = 1.41, 95% CI: 1.13–1.61), altered taste sensation (OR = 1.34, 95% CI: 1.02–1.62), sensitive gums (OR = 1.71, 95% CI: 1.13–1.47), and dental caries (OR = 1.32, 95% CI: 1.27–1.52) domains with *p* < 0.05. Those who had greater readiness to learn more (OR = 5.37, 95% CI: 4.21–7.78) and nurses and pharmacists, i.e., the profession, were associated with a positive attitude toward the prevention and management of dental/oral health problems of PC patients (Table 5). Nurses and pharmacists with less years of experience (OR = 1.31, 95% CI: 1.43–3.11) and those who had higher degrees (OR = 1.4, 95% CI: 1.47–2.27) also had a more positive attitude toward the prevention and management of dental and oral health problems of PC patients than did those with a lower qualification (*p* < 0.05).

## 4. Discussion

The part of the oral cavity is teeth and gums which allows us to speak, smile, and chew. Cavities (tooth decay), gum (periodontal) disease, and oral cancer are some of the most common diseases affecting our oral health. Oral health is included as one of the 23 leading health indicators in Healthy People 2030. According to the World Health Organization (WHO), oral health is an important component of overall health and quality of life, and improving people's oral health around the world can improve their quality of life [12]. Good lifestyle choices, earlier diagnosis, and improved access to dentists during the pandemic are some of the key challenges in combating dental or oral issues of postchemotherapy, according to the State of Mouth Cancer UK Report 2020/21 [13].

In general, treating malignant diseases affects the mouth both directly and indirectly through effects on immune function or other systemic side effects. Pain, mucositis, salivary gland dysfunction, changes in taste, infections, difficulty in swallowing, fibrosis, soft tissue and/or bone necrosis, exacerbation of dental and periodontal diseases, and recurrent or secondary malignancy are all common oral complications [14]. Cancer survival has improved in recent decades as a result of evolving etiologies and treatment advancements, and the population of cancer survivors continues to grow [15]. This growing number of survivors presents new challenges, particularly in the care of patients with complex medical, oral/dental, and psychosocial needs. Oral complications in this population typically necessitate multidisciplinary collaboration among various professionals and healthcare providers. Primary healthcare professionals, such as nurses, pharmacists, and lab technicians, are “change agents” at the community level, and can thus collaborate to effectively implement oral health promotion strategies, with a key role in early prevention and referral oral health services.

### 4.1. Knowledge

The current study found that PHPs had a low level of oral health knowledge, as most of them could identify only dental caries as the major issue of PC patients. Other dental or oral issues such as mucositis/mucosal pain, dysgeusia/hypogeusia/altered taste sensation, xerostomia/dry mouth/hyposalivation, jaw pain, etc. were least identified (Table 2). The overall level of knowledge on dental or oral health was better among nurses (64.56%), followed by pharmacists (54.5%); lab technicians scored less (30.03%) comparatively. However, pharmacists had better knowledge (65.2% vs 63.0% and 27.7%) on mucositis/mucosal pain among the PHPs (Table 3). Another study found similar results among primary care providers [16]. Wooten et al. reported that nurses have limited knowledge of oral health in a study of nurse practitioners' knowledge, opinions, and practice behaviors regarding oral disease and its outcomes [17].

Although the WHO recommends embedding oral health advancement with general preventive medicine and providing expert and policy support to nations to achieve this goal [18], our study found that primary healthcare providers receive limited training in this area [19, 20], notably in patients with postchemotherapy issues. This finding is also consistent with primary care healthcare professionals' lack of knowledge of pediatric OHC in a public healthcare setting in Tehran [16].

The WHO emphasized the importance of providing oral healthcare in developing countries as part of the primary healthcare programs [18]. During this COVID-19 pandemic, PHPs may be the only reliable source of oral health preventive care for PC patients, especially given the limited contact with oncology or dental professionals. PHPs can help diagnose oral health issues in their early stages and preclude their systemic effects in immunocompromised PC patients if they are given appropriate training in this field. According to studies, these PHPs have a significant impact on preventive activities and are a potential target for educational interventions [21–23].

### 4.2. Attitude

About the PHPs' attitude, most of them expressed positively toward their willingness to learn more and expressed the need for collaboration or telemedicine facility to manage the dental and oral health of PC patients. Nearly half of them had a negative opinion on handling PC patients' oral or dental health issues effectively (Figure 1). This may be due to the fact that they might have inadequate knowledge and practice experience of PC patients dental or oral health issues. The above attitude finding could be related to the PHPs' awareness of their lack of field knowledge. Our findings highlight the importance of adequate training and guidance for PHPs, a process that has proven effective to provide oral health promotion and disease prevention activities. Hence, primary healthcare professionals can make a team effort to effectively implement dental and oral health promotion approaches to PC patients.

### 4.3. Practice

Determining the practice as a part of empowerment of the PHPs toward the prevention and management of dental and oral health problems of PC patients reveals that though 18.81% of them had PC patients with dental or oral health issues in the past 3 months, Only 3.5% of the nurses and 0.8% of the pharmacists treated these patients' dental or oral issues often (Table 4). The results of a systemic review reported some empirical evidence that a dental health awareness campaign for care home nurses could enhance the nurses' oral healthcare knowledge and attitude [24]. If the PHPs' knowledge improves and gains positive attitude, this will enhance their practical skills as well. Because knowledge and attitude are directly interrelated to practice, there was a significant strong correlation between PHPs' knowledge and their attitude and practice [25–27].

### 4.4. Multivariate Analysis

The multivariate analysis shows that PHPs with higher knowledge scores, particularly in the mucositis/mucosal pain and dental domains, as well as those who were more willing to learn more about oral health had more positive attitudes toward the prevention and management of dental and oral health problems of PC patients (Table 5). Nurses and pharmacists with less years of experience and those who had higher degrees had a more positive attitude toward the prevention and management of dental and oral health problems of PC patients than did those with a lower qualification. Obtaining a higher degree may have improved their training, possibly due to their ability to search for the required information. In contrast to this study, the study by Rabiei S et al. [16] reported that nurses with a lower education (OR = 1.9) had a more positive attitude toward oral healthcare. Our findings call for training and continuing education for primary care providers about the oral health issues of PC patients, especially during this pandemic.

## 5. Clinical Relevance

### 5.1. Scientific Rationale for Study

PHPs not only play a role in the promotion of health, but also have a vital role in four levels of preventive care such as primordial, primary, secondary, and tertiary. In this scenario, information on the empowerment of PHPs in the dental/oral care of PC patients is still limited. In pandemic situations, especially in developing countries, the role of PHCs is important. Though their empowerment of knowledge in the dental/oral care of PC patients is a choice and cannot be ignored, this necessity provoked us to do the current study.

### 5.2. Principal Finding

The evidence for the role of PHPs in the oral care of PC patients is still limited in terms of knowledge, attitude, and practice on dental and oral health issues of PC patients.

### 5.3. Practical Implications

It is crucial that dental and oral health issues of PC patients be addressed during PHC visits to reduce the impact of these conditions on patients' morbidity, mortality, and quality of life, especially during this pandemic when there are limited points of referral or care. An educational intervention and continuous reinforcement on identification and management of these issues in collaboration with dental specialists, for the PHPs, can significantly reduce patients' agony, related morbidity as well as improve their quality of life.

## 6. Recommendations

- A similar study with larger samples in a wider population can be done
- To improve the knowledge and skills of the PHPs; educational or training sessions with recent updates on research should be done to improve the quality of life of PC patients

## 7. Conclusion

Our study found that PHPs had limited empowerment with low level of knowledge in dental or oral health issues of PC patients while also displaying a generally positive attitude and a noteworthy readiness to obtain more training in this field. The findings of our study highlight the critical need for appropriate training and encouragement, particularly among PHPs working in PHCs, to promote oral health and provide appropriate conditions/care for PC patients.

## Figures and Tables

**Table 1:** Demographic data of the PHPs.

Variables	No.	%

Age (years)		
20–25	51	23.4
26–30	40	18.3
31–35	69	31.7
>36	58	26.6
Sex		
Male	80	36.7
Female	138	63.3
Work experience		
Less than 1 year	84	38.5
1–3 years	77	35.3
4–6 years	37	17.0
7–9 years	11	5.1
≥ 10 years	9	4.1
Profession		
Nurses	112	51.4
Pharmacists	71	32.6
Lab technicians (LTs)	35	16.1
Level of education		
Diploma	105	48.2
Bachelor's degree	99	45.4
Master's degree	14	6.4
Approximate number of patients per day		
<10	51	23.4
10–50	117	53.7
51–100	42	19.3
>100	8	3.7
Do you get any patients with postchemotherapy treatment?		
Yes	48	22.0
No	170	78.0
If yes, did any of them attend the PHC with dental or oral health problems in the past 3 months?		
Yes	41	18.8
No	177	81.2

**Table 2:** Identification of dental- and oral-related health issues among PC patients.

S. no.	Dental/oral problems of PC patients	Yes	%

1	Mucositis/mucosal pain	137	62.84
2	Dysgeusia/hypogeusia/altered taste sensation	116	53.21
3	Xerostomia/dry mouth/hyposalivation	142	65.14
4	Sensitive gums	153	70.18
5	Dental caries	187	85.78
6	Oral candidosis	121	55.51
7	Bacterial sialadenitis	89	40.83
8	Cachexia	193	88.53
9	Trismus/fibrosis	94	43.12
10	Mastication/jaw pain	57	26.15

**Table 3:** Level of knowledge of PHPs on dental and oral health among postchemotherapy patients.

Knowledge	Nurses (%)	Pharmacists (%)	Lab technicians (%)

Mucositis/mucosal pain	63.0	65.2	27.7
Dysgeusia/hypogeusia/altered taste sensation	63.3	41.8	21.37
Xerostomia/dry mouth/hyposalivation	69.1	61.4	19.1
Sensitive gums	66.4	51.8	38.8
Dental caries	61.0	52.3	43.2
Overall knowledge	64.56	54.5	30.03

**Table 4:** Practice of PHPs toward prevention and management of dental and oral health problems of PC patients.

Statement of practice	Never (%)			Rarely (%)			Sometimes (%)			Often (%)			Always (%)		
	Nurses	Pharmacists	LTs	Nurses	Pharmacists	LTs	Nurses	Pharmacists	LTs	Nurses	Pharmacists	LTs	Nurses	Pharmacists	LTs

Have ever identified or diagnosed the dental or oral health problems of PC patients?	38.4	69.0	88.6	11.6	11.3	5.7	29.5	12.0	4.2	18.8	2.8	0	1.8	0	0
Have ever treated the dental or oral health problems of PC patients?	86.6	94.4	97.1	9.8	11.3	0	3.6	2.8	2.9	0	0	0	0	0	0
Have ever treated or given advise to prevent dry mouth to PC patients?	75.0	88.7	85.7	8.0	8.5	11.4	15.2	2.8	2.9	1.8	0	0	0	0	0
Have ever treated or given advise to prevent the loss of taste to PC patients?	83.0	97.2	94.3	6.3	0	0	8.0	2.8	5.7	2.7	0	0	0	0	0
Have ever treated or given advise to prevent mucositis to PC patients?	63.4	73.2	82.9	29.5	15.5	11.4	7.1	12.7	5.7	0	0	0	0	0	0
Have ever treated or given advise to prevent sensitive gums to PC patients?	36.6	36.7	94.3	47.3	15.5	5.7	10.7	43.7	0	0	2.7	0	0	0	0
Have ever treated or given advise to prevent dental caries to PC patients?	73.2	40.9	82.9	15.2	43.7	11.4	9.8	15.5	5.7	1.8	0	0	0	0	0

**Table 5:** Factors associated with PHPs' attitudes (positive/negative) toward the prevention and management of dental/oral health problems of PC patients controlling the demographic data by a logistic regression model.

	B.	S.E.	Sig	OR	95% CI for OR	
					Lower	Upper

Mucositis/Mucosal pain	0.21	0.65	0.021	1.41	1.13	1.61
Altered taste sensation	0.31	0.07	0.000	1.34	1.02	1.26
Xerostomia/dry mouth/hyposalivation	0.09	0.04	0.173	1.03	0.91	1.31
Sensitive gums	0.13	0.06	0.023	1.17	1.13	1.47
Dental caries	0.16	0.21	0.05	1.32	1.27	1.51
Profession	0.7	0.31	0.000	1.59	1.09	2.43
Readiness to learn	1.970	0.22	0.000	5.37	4.21	7.78
Qualification	0.643	0.29	0.000	1.81	1.39	2.74

## Data Availability

The data presented in this study are available on request from the corresponding author.
