# Corrigendum to “Empowerment of Primary Healthcare Providers on the Prevention and Management of Dental or Oral Health Issues among Postchemotherapy Patients in Pandemic”

**DOI:** 10.1155/2024/9876360

**Published:** 2024-12-02

**Authors:** Premalatha Paulsamym, Krishnaraju Venkatesan, Jamal Moideen Muthu Mohamed, ShadiaHamoud Alshahrani, Ramasubbamma Ramaiah, Vigneshwaran Easwaran, Mohamed El-Sherbiny, Mohamed Dosoky, Noohu Abdulla Khan, Kousalya Prabahar, Geetha Kandasamy, Kibebe Sahile

**Affiliations:** ^1^College of Nursing, Mahalah Branch for Girls King Khalid University, Abha, Asir, Saudi Arabia; ^2^Department of Pharmacology, College of Pharmacy, King Khalid University, Abha, Asir, Saudi Arabia; ^3^College of Pharmacy, Shri Indra Ganesan Institute of Medical Science, Tiruchirappalli 620012, Tamil Nadu, India; ^4^Department of Clinical Pharmacy, College of Pharmacy, King Khalid University, Abha, Saudi Arabia; ^5^Department of Basic Medical Sciences, College of Medicine, AlMaarefa University 71666, Riyadh, Saudi Arabia; ^6^Department of Neuroscience Technology-College of Applied Sciences, Jubail Imam Abdulraman Bin Faisal University, Al Jubail, Saudi Arabia; ^7^Department of Pharmacy Practice, Faculty of Pharmacy, University of Tabuk, Tabuk 71491, Saudi Arabia; ^8^Department of Chemical Engineering, College of Biological and Chemical Engineering, Addis Ababa Science and Technology University, Addis Ababa, Ethiopia

In the article titled “Empowerment of Primary Healthcare Providers on the Prevention and Management of Dental or Oral Health Issues among Postchemotherapy Patients in Pandemic” [1], there was an error in the acknowledgment section. The last statement has been removed, and the corrected section appears below:

## References

[1] Paulsamym P., Venkatesan K., Muthu Mohamed J. M (2022). Empowerment of Primary Healthcare Providers on the Prevention and Management of Dental or Oral Health Issues among Postchemotherapy Patients in Pandemic. *Journal of Healthcare Engineering*.

